# Minimally invasive resection rectopexy as a treatment method for obstructive defecation (ODS): functional outcome in ODS; constipation and fecal incontinence

**DOI:** 10.1186/s12893-026-03560-5

**Published:** 2026-03-05

**Authors:** Jamal Driouch, Lara Schlaffke, Shazadi Sajid, Dirk Bausch, Omar Thaher

**Affiliations:** 1 Department of Surgery, Elisabeth Hospital, Lehrkrankenhaus of Ruhr University of Bochum, Hochstraße 63, Iserlohn, 58638 Germany; 2 Department Medical Engineering, FH Dortmund, University of Applied Sciences and Arts, Dortmund, Germany; 3 Department of Surgery, Marien Hospital Herne, Ruhr University of Bochum, Hölkeskampring 40, Herne, 44625 Germany

**Keywords:** Laparoscopic resection rectopexy, Obstructive defecation, Fecal incontinence, Constipation, Left colon resection, Natural-Orifice-Specimen-Extraction, Intracorporeal anastomosis, Robotic-NOSE

## Abstract

**Background:**

Minimally invasive resection rectopexy is an effective treatment for obstructive defecation syndrome (ODS) in carefully selected patients, offering favorable functional outcomes with potentially reduced surgical trauma. As minimally invasive techniques continue to advance in colorectal surgery, integrating resection rectopexy with Natural Orifice Specimen Extraction (NOSE) or minilaparotomy retrieval may further enhance postoperative bowel function by minimizing constipation and fecal incontinence. We hypothesize that surgical outcomes are influenced by the severity of disease.

**Methods:**

Between January 2019 and June 2022, 85 patients with ODS underwent minimally invasive resection rectopexy. Patient characteristics were assessed using standardized questionnaires. Surgical outcomes, complications, and patient satisfaction were evaluated at 1, 3, and 6 months postoperatively. Symptom severity was quantified using the Wexner Constipation Score (WCS), Wexner Incontinence Score (WIS), and Altomare Obstructive Defecation Syndrome (AOS) Score. Repeated measures ANOVA was performed to assess longitudinal changes in these measures over time.

**Results:**

All procedures were successfully completed laparoscopically without conversion. The mean patient age was 58.5 years (SD = 16.5). Complications were classified as Clavien–Dindo grade IIIa (*n* = 3), IIIb (*n *= 8), and IVa (*n *= 1). Repeated measures ANOVA demonstrated significant postoperative improvements in WCS (*p *< 0.0001) and AOS (*p *< 0.0001). Although WIS changes were not statistically significant, a gender-specific effect was observed (*p *= 0.011). Post hoc analyses revealed no significant temporal effects for WIS across genders. Follow-up was limited to 6 months.

**Conclusion:**

Minimally invasive resection rectopexy provides substantial symptom relief, particularly in reducing constipation and obstructive defecation. While outcomes for fecal incontinence remain variable, the procedure seems safe, feasible, and markedly improves the quality of life in patients with ODS. Long-term outcomes and recurrence rates require further study.

**Supplementary Information:**

The online version contains supplementary material available at 10.1186/s12893-026-03560-5.

## Introduction

Obstructive Defecation Syndrome (ODS) is a common condition resulting from functional or mechanical bowel dysfunction caused by morphological alterations of the distal colon and rectum [1, 2]. It is characterized by impaired defecation, including fragmented stools, excessive straining, a sensation of incomplete evacuation, tenesmus, urgency, pelvic heaviness, the need for digital assistance or enemas, as well as bleeding and pain [3].

Surgical intervention is indicated when subjective symptoms are accompanied by morphological changes in the rectosigmoid or rectal region, such as dolichosigmoid with sigmoidocele, cul-de-sac syndrome, external or internal rectal prolapse, sigmoid stricture with advanced intussusception, and associated pelvic floor dysfunction [4, 5].

Various surgical approaches have been described, including transanal, transvaginal, transperineal, and abdominal techniques, performed either laparoscopically or robotically [6–8]. Morphological alterations can lead to both proximal and distal outlet obstruction involving the left colon and rectum, and many patients present with a combination of both.

An abdominal approach is typically preferred for proximal obstructions, such as dolichosigmoid with sigmoidocele, cul-de-sac syndrome, or sigmoid stricture with advanced intussusception. Conversely, transanal or transvaginal procedures are more suitable for distal outlet obstruction [9, 10]. To date, no standardized operative pathway for the surgical management of ODS has been established [3].

To address both proximal outlet obstruction (via rectosigmoid resection) and distal obstruction (via suture rectopexy) in a single operation, the resection rectopexy was developed [11–13]. Using the collected demographic and surgical data, we evaluated postoperative functional outcomes, hypothesizing that surgery leads to significant functional improvement and that preoperative function is associated with postoperative results.

## Materials and methods

In this study, minimally invasive resection rectopexy with ventrolateral peritoneal suture pexy was performed in 85 patients diagnosed with obstructive defecation syndrome (ODS). Between January 2019 and June 2022, 28 patients underwent laparoscopic resection rectopexy with specimen retrieval via mini-laparotomy (Lap-LRR), 34 underwent robot-assisted Natural Orifice Specimen Extraction- resection rectopexy (NOSE-RRR), and 23 underwent NOSE-assisted laparoscopic resection rectopexy (NOSE-LRR).

### Patient selection criteria

Morphological criteria for ODS indicating the need for minimally invasive surgery included: dolichocolon with cul-de-sac syndrome or sigmoidocele, sigmoid kinking or stenosis with advanced intussusception (grade II–III), dolichocolon with intussusception, rectal prolapse (grade I–III), and combinations of rectal prolapse with descending perineum.

Exclusion criteria comprised purely functional disorders such as slow-transit constipation or irritable bowel syndrome, previous sigmoid resections, high comorbidities associated with increased anesthetic risk, prior pelvic floor reconstruction, or pelvic radiation therapy. Redo surgeries were also excluded.

### Surgical procedure selection

Patients were allocated to one of three surgical approaches “Lap-LRR, NOSE-LRR, or NOSE-RRR” based on cohort assignment and surgical considerations, with approximately equal numbers (*n* ≈ 40) per cohort. Remaining patients fulfilling inclusion criteria were incorporated into the study. The NOSE-RRR cohort was enrolled last, after robotic-assisted surgery techniques had been optimized.

The choice between suture rectopexy and mesh rectopexy during resection rectopexy was deliberate. Mesh was avoided due to concerns regarding bacterial contamination associated with simultaneous intracorporeal anastomosis, whereas suture rectopexy was considered safer in this context.

### Data collection and outcome assessment

All data were prospectively collected and analyzed. Patient- and disease-specific characteristics were assessed through clinical examination and standardized questionnaires. Surgical and functional outcomes, operative time, length of hospital stay, postoperative complications, and patient-reported satisfaction were evaluated at 1, 3, and 6 months postoperatively.

The questionnaire used in the investigation to assess perioperative and postoperative outcomes was particularly designed for the study and has not been used in any other studies in the same manner.

Symptom severity and functional outcomes were quantified using the Wexner Constipation Score (WCS), Wexner Incontinence Score (WIS), and Altomare Obstructive Defecation Syndrome (AOS) Score. These questionnaires are standardized and validated instruments, as referenced in the manuscript.

Data were collected preoperatively and postoperatively at defined intervals using questionnaires and direct patient interviews during clinical visits. All patients underwent structured follow-up in our outpatient clinic at 1, 3, and 6 months after surgery, including proctological examination and disease-specific evaluation.

### Statistical analysis

Based on the correlations observed between repeated measurements in our dataset, we conducted a post-hoc power analysis using the statsmodels package in Python. For each outcome measure (ODS, Incontinence, Constipation), within-subject correlations between time points were calculated and used to adjust the effect size (Cohen’s f) for repeated measures designs. The resulting required sample sizes for achieving 80% power at α = 0.05 ranged from 43 participants (Incontinence) to 113 participants (ODS). Our study included 85 participants with complete follow-up data, which exceeds the requirement for Incontinence and Constipation analyses and is close to the requirement for ODS. Given the strong within-subject correlations and the balanced design, the achieved power is sufficient to detect clinically relevant effects in our primary analyses. One participant had missing follow-up data and was excluded from the repeated measures analyses. However, this case was included in baseline descriptive statistics where applicable. No imputation was performed. Sensitivity checks confirmed that the excluded participant did not differ meaningfully from the rest of the cohort in baseline characteristics, suggesting that the missing data were random and unlikely to introduce bias.

All statistical analyses were conducted using R (version 4.3.2) and RStudio (version 2024.09.0). A repeated measures ANOVA was performed using the `aov()` function to assess the effect of time on the functional outcome variables AOS, WIS and WCS across time points (preoperative baseline, 1, 3 and 6 months). In the case of significant effects of time, pairwise comparisons were made using the Bonferroni correction (`pairwise.t.test ()`). The significance level was set at α = 0.05. Data visualization was performed with `ggplot2` (version 3.4.4), and descriptive statistics were calculated using `dplyr` (version 1.1.4). To reveal predictive values of baseline scores, correlation analysis were performed between baseline scores and differences between baseline and 6 months follow up, using `cor.test()`. All statistical tests were two-tailed. To verify assumptions of the repeated-measures ANOVA, sphericity was tested with Mauchly’s test using the ezANOVA function (ez package). In cases where the sphericity assumption was violated, Greenhouse–Geisser correction was applied.

### Diagnostic work-up

During the initial proctological consultation, a comprehensive medical history was obtained, including a detailed description of presenting symptoms. A standardized proctological examination was performed during the same session and included inspection, digital rectal examination, anal manometry, endoanal ultrasound, proctoscopy, and rectoscopy.

In cases where endoscopic evaluation revealed high-grade intussusception (grade II–III), rectal prolapse, or pelvic floor descent associated with symptoms typical of outlet obstruction, further diagnostic investigations were performed. These included contrast enema, dynamic imaging such as X-ray or MR defecography, and assessment of colonic transit time.

When a family history of colorectal stenosis or tumors was present, colonoscopy was additionally indicated. All patients underwent standard bowel preparation on the day before surgery, followed by administration of an enema immediately prior to the operation.

### Surgical technique

All procedures were performed under general anesthesia with standard perioperative antibiotic prophylaxis using cefuroxime and metronidazole, unless contraindicated, in which case appropriate alternatives were administered. All patients underwent standard bowel preparation before surgery and rectal lavage immediately prior to the procedure. Patients were positioned in the Lloyd–Davies position.

The stapler size was preoperatively estimated using rectoscopy, by assessing rectal elasticity and ruling out stenoses. Intraoperatively, the bowel lumen was calibrated using anal bougies of increasing diameter under laparoscopic visualization to ensure optimal stapler selection and tension-free anastomosis.

### Laparoscopic resection rectopexy (Lap-LRR)

After standard mobilization of the sigmoid and rectum within the Waldeyer and Denonvilliers layers, resection was performed with the distal margin at the mid-rectum and the proximal margin at the sigmoid–descending junction, generally defined as the transition from the retroperitoneally fixed descending colon to the mobile sigmoid colon, approximately at the level of the iliac crest or pelvic inlet. This anatomical boundary may vary slightly between patients. The specimen was retrieved via a lateral mini-laparotomy. The double-stapling-anastomosis was created intracorporeally using a circular stapler introduced transanally under laparoscopic visualization, followed by a routine leak test. The peritoneal fold was reconstructed with a continuous barbed suture and interrupted ventral fixation to the rectal wall. Rectopexy was completed in a laterodorsal fashion. A pelvic drain was placed before fascial and skin closure.

### NOSE-assisted procedures (NOSE-LRR / NOSE-RRR)

In NOSE-assisted techniques, specimen extraction was performed transanally following complete laparoscopic or robotic mobilization of the sigmoid and rectum. The rectum was divided at the mid-level, and the specimen was delivered through the anus using grasping forceps under laparoscopic control. After extraction, the anvil (pressure plate) was introduced transanally and positioned within the proximal colon. The bowel was then divided with an Endo-GIA stapler below the anvil to complete specimen removal. The rectal stump was resected with the Endo-GIA, and the anvil was pulled through the proximal colon stump.

The circular stapler was introduced transanally, and the end-to-end colorectal double-stapling- anastomosis was performed under direct visualization. The peritoneal fold was reconstructed as in the Lap-LRR group, followed by laterodorsal rectopexy. A drain was placed in the small pelvis, and closure was performed in layers. All procedures were completed laparoscopically or robotically without conversion.

### Postoperative stage and follow-up

As part of our established extended recovery program, patients were mobilized early, initially at the edge of the bed, and gradually advanced from a liquid diet to a full diet as tolerated. To reduce the risk of pelvic infections following transanal specimen extraction, antibiotic therapy was continued for three days postoperatively.

Patients received daily guidance from physical therapy to support mobilization until discharge. Discharge criteria included: tolerance of a complete diet, return of bowel function, independent ward-level mobilization, daily progress in self-care, and normalization or decline of inflammatory laboratory parameters.

Patients were advised to avoid foods high in cereals or those causing excessive flatulence for 14 days and to maintain adequate hydration. Structured clinical follow-ups, including physical examination and symptom assessment, were conducted at 1, 3, and 6 months postoperatively.

## Results

The study included 85 patients, of whom 18 were male (21%) and 67 were female (79%). The mean age was 58.5 years (SD: 16.5), and the mean body mass index (BMI) was 26.9 (SD: 5.0). A substantial proportion of patients (*n* = 70, 82.4%) had a history of previous abdominal or pelvic surgery. Among these, 36 patients (42.4%) had undergone one or two prior procedures, while 34 patients (40.0%) had undergone three or more.

Preoperative health status according to the American Society of Anesthesiologists (ASA) classification was as follows: ASA 1, 12 patients (14.1%); ASA 2, 47 patients (55.3%); and ASA 3, 26 patients (30.6%).

The primary presenting symptom was constipation, reported by 74 patients (87.1%). Intussusception was observed in 22 patients (25.9%) with Grade II and 63 patients (74.1%) with Grade III. Rectocele was present in 49 patients (57.6%), and rectal prolapse was classified as Grade I in 7 patients (8.2%), Grade II in 2 patients (2.4%), and Grade III in 3 patients (3.5%). Regarding fecal incontinence, 15 patients (17.6%) reported Grade I, 30 patients (35.3%) Grade II, and 31 patients (36.5%) reported no incontinence.

Comorbidities included Type 2 diabetes mellitus in 20 patients (23.5%) and Type 1 diabetes mellitus in 1 patient (1.8%). Hypertension was reported in 49 patients (57.6%). All demographic and comorbidity data are summarized in Table 1.

**Table 1:** Baseline demographic and clinical characteristics of patients included in the study

	all (*n* = 85)		all (*n* = 85)
Gender		Incontinence	76 (89,4%)
		0	31 (36,5%)
male	18 (21%)	I°	15 (17,6%)
female	67 (79%)	II°	30 (35,3%)
		Prolaps	12 (14,1%)
Age in years *(mean)	58,5 (SD 16,5)	I°	7 (8,2%)
		II°	2 (2,4%)
		III°	3 (3,5%)
BMI (mean)	26,9 (SD 5,0)	Rectozele	49 (57,6%)
ASA		Intussuszeption	85 (100%)
I	12 (14.1%)	I°	0
II	47 (55,3%)	II°	22 (25,9%)
III	26 (30,6%)	III°	63 (74,1%)
Anastomotic leak	1 (1,2%)	Constipation	74 (87,1%)
Previous abd. / rect. op`s		Diabetes	21 (24,7%)
		Typ 1	1 (1,8%)
		Typ 2	20 (23,5%)
0	15 (17,6%)		
1–2	36 (42,4%)		
>=3	34 (40%)		
Anastomotic stenosis	3 (3,5%)	Art. Hypertonie	49 (57,6%)
Clavien-Dindo		Postoperative hemorrhage	
		abdominal	2 (2,4%)
IIIa	3 (3,5%)	anal	2 (2,4%)
IIIb	8 (9,4%)		
IVa	1 (1,2%)		

### Surgical results

Minimally invasive resection rectopexy was successfully performed in all study groups: the laparoscopic group with specimen retrieval via mini-laparotomy (Lap-LRR) and the transanal specimen extraction groups (NOSE-LRR and NOSE-RRR), with no conversions to open surgery. Transanal specimen extraction and intracorporeal stapled anastomoses were successfully completed in all NOSE-LRR and NOSE-RRR patients. Blood loss was minimal in all cases, with < 50 mL reported per procedure.

Overall, 12 complications (14.1%) were observed. These included 3 complications (3.5%) classified as Clavien–Dindo IIIa and 8 complications (9.4%) classified as IIIb, requiring reoperation. One patient (1.2%) experienced an anastomotic leak with concomitant ureter injury. Subjective patient satisfaction was high, with 77 patients (90.6%) reporting satisfaction with their surgical outcomes.

To compare postoperative outcomes between surgical techniques (NOSE-RRR, NOSE-LRR, and Lap-LRR), complication rates were analyzed using Chi-square and Fisher’s Exact Test, accounting for the relatively small sample sizes and the categorical nature of the data. Postoperative complications are summarized in Table 2.

**Table 2:** Postoperative complications according to surgical technique with global and pairwise significance levels

Complication	NOSE-RRR (*n* = 34)	NOSE-LRR(*n* = 23)	Lap-LRR(*n* = 28)	Total (*n* = 85)	*p*-value (global)	Significant pairwise comparisons
Wound healing disorder	12.9% (1)	00.0% (0)	7.1% (2)	3.5% (3)	0.377	–
Anastomotic leakage	0.0% (0)	0.0% (0)	3.6% (1)	1.2% (1)	0.357	–
Clavien IIIa	0.0% (0)	13.0% (3)	0.0% (0)	3.5% (3)	0.015	trend (NOSE-LRR vs. others, *p* ≈ 0.06–0.09)
Clavien IIIb	2.9% (1)	0.0% (0)	25.0% (7)	9.4% (8)	0.002	Lap-LRR vs. NOSE-RRR (*p* = 0.018); Lap-LRR vs. NOSE-LRR (*p* = 0.012)
Clavien IVa	0.0% (0)	0.0% (0)	3.6% (1)	1.2% (1)	0.357	–
Conversion	0.0% (0)	0.0% (0)	0.0% (0)	0.0% (0)	1.000	–
Re-operation	2.9% (1)	0.0% (0)	28.6% (8)	10.6% (9)	0.001	Lap-LRR vs. NOSE-RRR (*p* = 0.008); Lap-LRR vs. NOSE-LRR (*p* = 0.006)

Wound healing disorders occurred in 2.9% (1/34) of NOSE-RRR patients and 7.1% (2/28) of Lap-LRR patients, while no cases were observed in the NOSE-LRR group (*p* = 0.377). Anastomotic leakage was rare, detected in 1 patient (3.6%) in the Lap-LRR group (*p* = 0.357). Clavien–Dindo IIIa complications were reported in 13.0% (3/23) of NOSE-LRR patients, with no events in the other two groups; this difference showed a trend but did not reach statistical significance (*p* = 0.015).

In contrast, Clavien–Dindo IIIb complications were significantly more frequent in the Lap-LRR group (25.0%, 7/28) compared to NOSE-RRR (2.9%, 1/34) and NOSE-LRR (0%, 0/23) (*p* = 0.002). Reoperations were required in 28.6% (8/28) of Lap-LRR patients, versus 2.9% (1/34) in NOSE-RRR and 0% in NOSE-LRR (*p* = 0.001). No significant differences were observed for Clavien–Dindo IVa complications (3.6% [1/28] in Lap-LRR vs. 0% in both NOSE groups; *p* = 0.357), and no conversions occurred in any group.

### Functional results

**Fig. 1:**
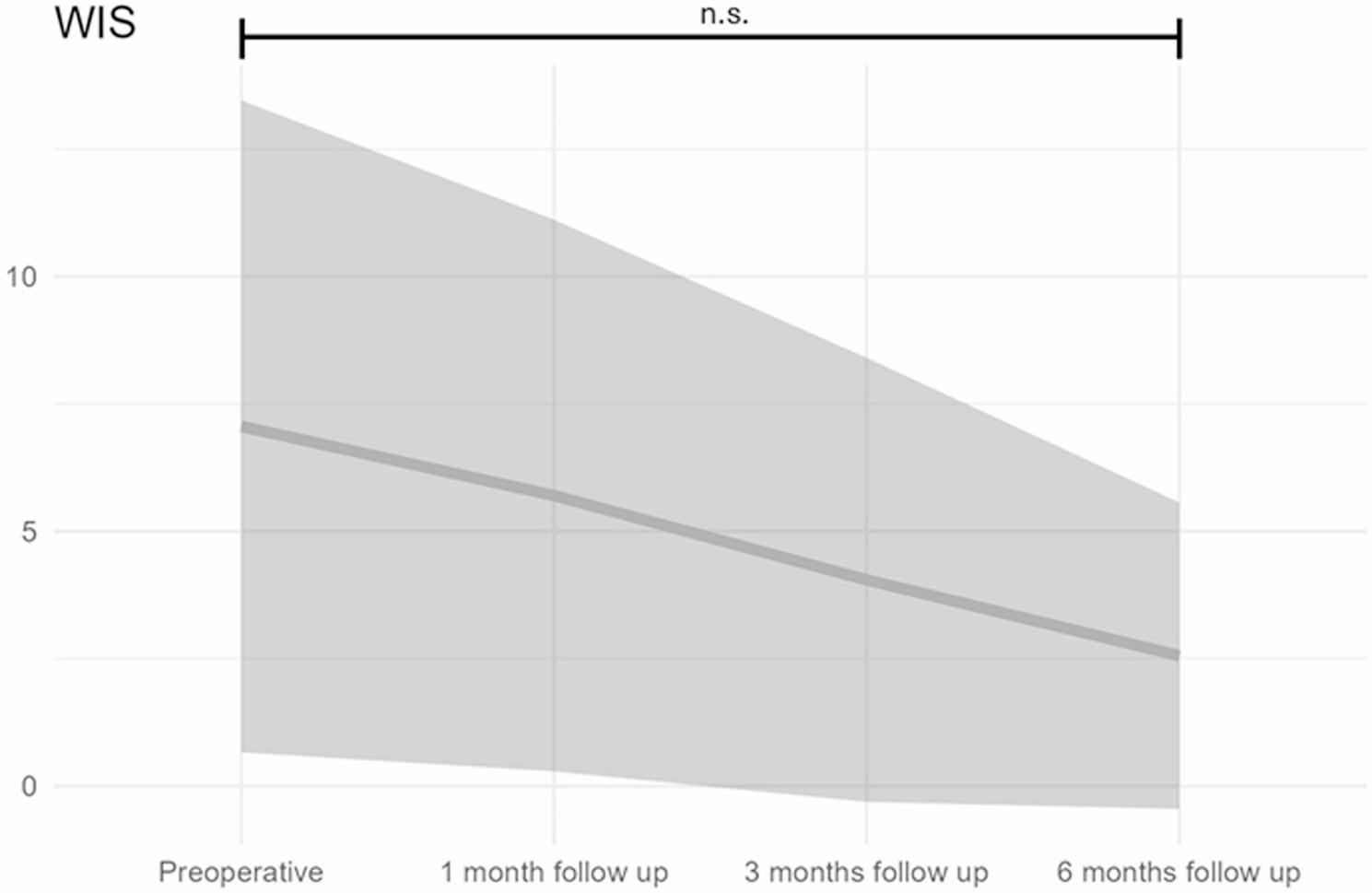
Progression of incontinence scores over different measurement time points (solid line = mean, grey area = standard deviation

The mean Wexner Incontinence Score (WIS) was 7.06 ± 6.4 preoperatively and decreased to 5.7 ± 5.4 at 1 month, 4.05 ± 4.36 at 3 months, and 2.56 ± 3.01 at 6 months postoperatively (Fig. 1). Although a positive trend was observed over time, the main effect of time did not reach statistical significance (F(3,325) = 1.76, *p* = 0.1546).

**Fig. 2:**
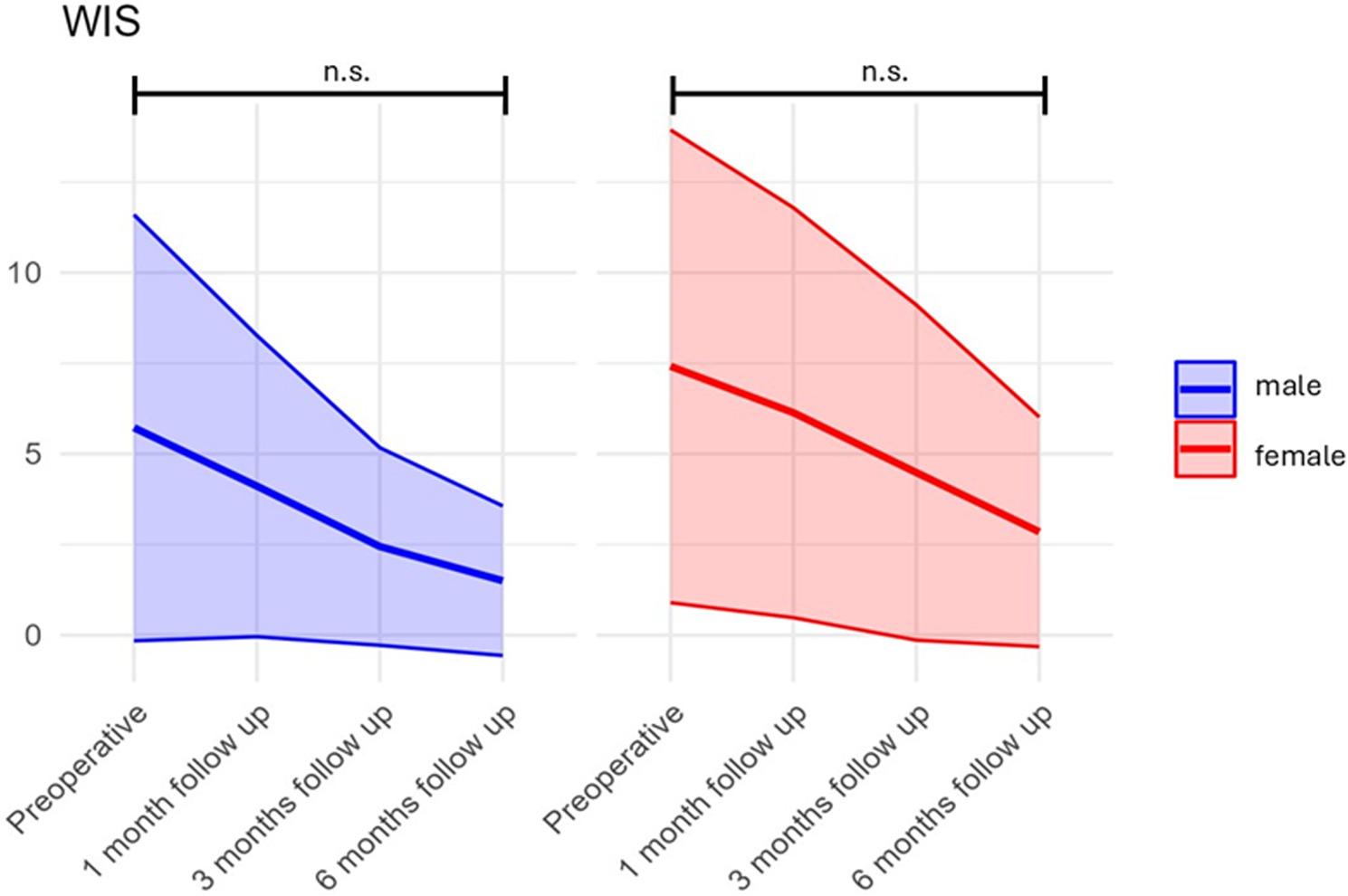
Progression of incontinence scores over different measurement time Points by Gender. The line plots show the mean incontinence scores for males (blue) and females (red) and the standard deviations

A significant main effect of gender was detected (F(1,325) = 6.61, *p* = 0.0106). Post hoc repeated-measures analyses conducted separately for males and females did not reach significance (time effect: F(3,64) = 0.99, *p* = 0.403 for males; F(3,257) = 1.05, *p* = 0.372 for females) (Fig. 2).

Repeated-measures ANOVA for WIS also revealed a significant main effect of surgical procedure (F(2,325) = 11.813, *p* < 0.001), indicating that the type of surgery influenced overall continence outcomes. However, the interaction between procedure and time was not significant (F(6,325) = 0.459, *p* = 0.711), suggesting that improvements over time were similar across surgical groups.

**Fig. 3:**
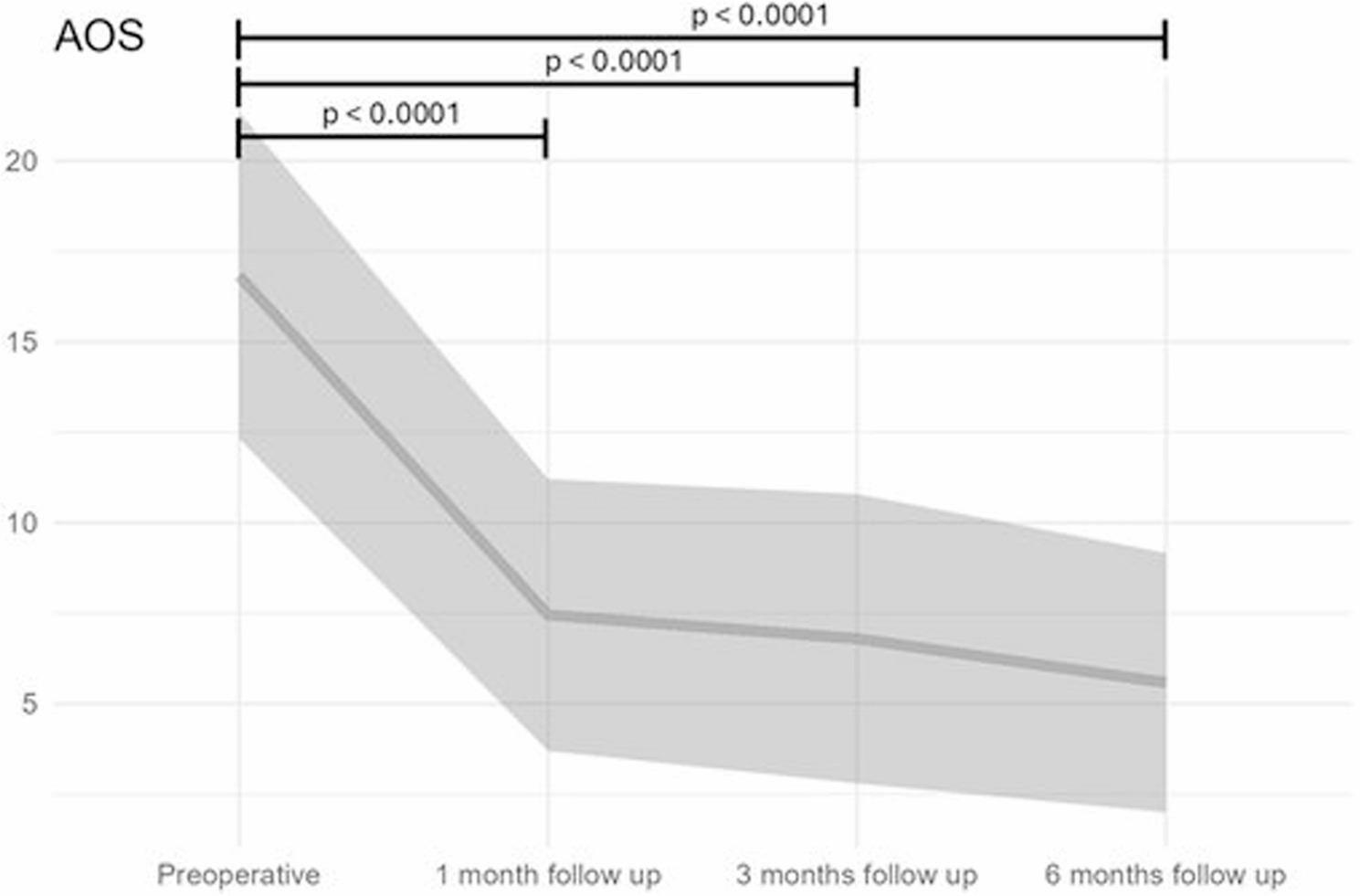
Progression of AOS pre and post surgery. Highly significant improvement was observed after surgery and remained after 6 months

The mean preoperative Altomare Obstructive Defecation Syndrome (AOS) score was 16.82 ± 4.51. Postoperatively, the scores decreased to 7.45 ± 3.76 at 1 month, 6.8 ± 3.4 at 3 months, and 5.58 ± 3.58 at 6 months, demonstrating a significant improvement after surgery (Fig. 3).

Repeated-measures ANOVA revealed a significant main effect of gender (F(2,325) = 5.30, *p* = 0.022), indicating differences between male and female patients. A significant main effect of time was also observed (F(3,325) = 35.91, *p* < 2 × 10⁻¹⁶), demonstrating significant changes in scores across the postoperative follow-up. The interaction between gender and time was not significant (F(3,325) = 0.168, *p* = 0.918), indicating that the effect of gender did not vary over time. Therefore, post hoc tests stratified by gender were not performed.

Post hoc paired t-tests confirmed highly significant improvements at 1, 3, and 6 months (all *p* < 0.0001). When stratified by surgical procedure, both procedure type (F(2,325) = 5.623, *p* = 0.018) and time (F(3,325) = 36.142, *p* < 0.001) had significant effects, while the interaction between procedure and time was not significant (F(6,325) = 0.993, *p* = 0.396), indicating that improvements over time were consistent across procedures.

**Fig. 4:**
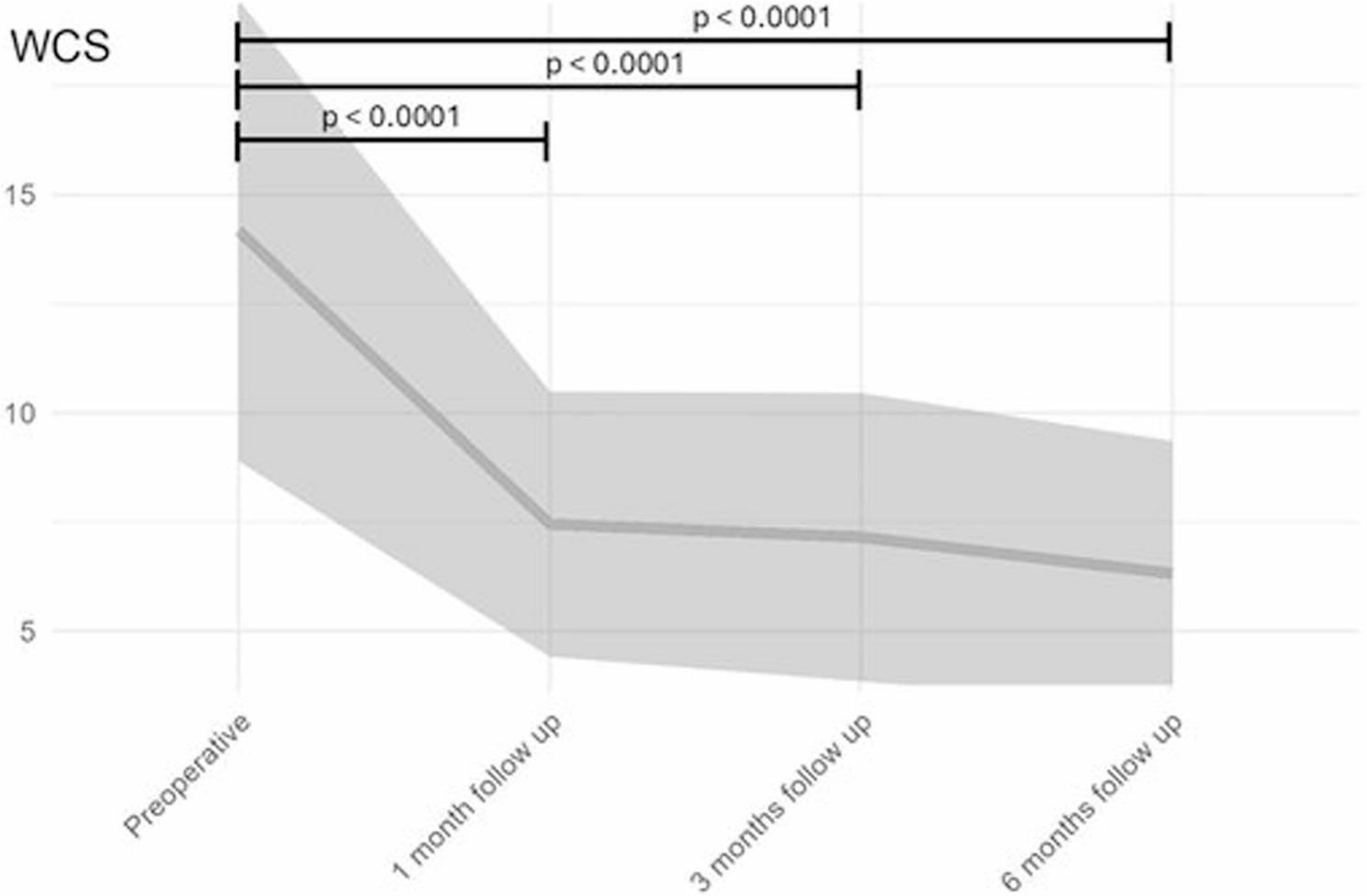
Progression of WCS. Highly significant improvement was observed after surgery and remained after 6 months

The Wexner Constipation Score (WCS), used as an additional functional parameter, also demonstrated significant improvement at 1, 3, and 6 months postoperatively. Preoperatively, the mean WCS was 14.18 ± 5.29, which decreased to 7.45 ± 3.04 at 1 month, 7.15 ± 3.01 at 3 months, and 6.32 ± 3.04 at 6 months (Fig. 4).

Repeated-measures ANOVA showed no significant main effect of gender (F(2,325) = 0.693, *p* = 0.406), indicating no differences in scores between males and females. A significant main effect of time was observed (F(3,325) = 21.924, *p* < 5.91 × 10⁻¹³), demonstrating significant improvement across follow-up periods. The interaction between gender and time was not significant (F(3,325) = 0.342, *p* = 0.795), indicating that gender did not influence changes over time; thus, post hoc tests stratified by gender were not performed.

Post hoc paired t-tests confirmed highly significant improvements at 1, 3, and 6 months (all *p* < 0.0001). When stratified by surgical procedure, time remained significant (F(3,325) = 21.889, *p* < 0.001), whereas procedure type (F(2,325) = 0.233, *p* = 0.630) and the interaction between procedure and time (F(3,325) = 0.007, *p* = 0.999) were not significant, indicating consistent improvement across surgical approaches.

**Fig. 5:**
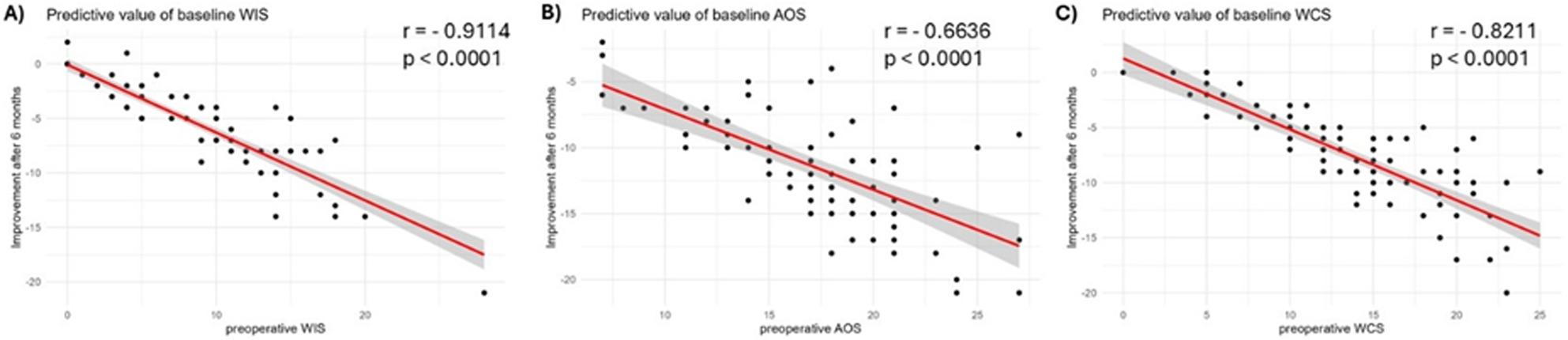
Correlation analysis of baseline values and delta between baseline and 6 months follow up to investigate the predictive value of baseline outcome measures. **A** predictive value of WIS, **B** predictive value of AOS, **C** predictive value of WCS

To evaluate the predictive value of preoperative functional status, Pearson correlation analyses were conducted between baseline scores and the changes observed at 6 months postoperatively (Δ = 6-month follow-up – baseline) for the Wexner Incontinence Score (WIS), Altomare Obstructive Defecation Syndrome (AOS) score, and Wexner Constipation Score (WCS) (Fig. 5).

For the Wexner Incontinence Score (WIS), Pearson correlation analysis revealed a strong negative correlation between baseline scores and the change from baseline to 6 months (*r* = − 0.911, t (82) = − 20.05, *p* < 2.2 × 10⁻¹⁶). This indicates that higher baseline WIS values were associated with greater postoperative improvement (reflected by a larger negative Δ; Fig. 5A).

For the Altomare Obstructive Defecation Syndrome (AOS) score, a moderate negative correlation was observed (*r* = − 0.664, t (82) = − 8.03, *p* = 6.03 × 10⁻¹²), suggesting that patients with higher baseline AOS scores experienced proportionally greater improvement at 6 months (Fig. 5B).

For the Wexner Constipation Score (WCS), a strong negative correlation was also found (*r* = − 0.821, t(82) = − 13.03, *p* < 2.2 × 10⁻¹⁶), indicating that higher preoperative WCS values were associated with significantly greater postoperative improvements (Fig. 5C).

## Discussion

Surgical treatment for dolichosigma and rectal prolapse, particularly via rectal resection and rectopexy, is widely used for patients with functional disorders such as chronic constipation, obstructive defecation, and fecal incontinence. Over recent years, several studies have examined the long-term functional outcomes of these procedures [14–16].

In the present study, we observed marked improvement in obstructive defecation syndrome (ODS) at 1, 3, and 6 months, as measured by the Wexner Constipation Score (WCS), Wexner Incontinence Score (WIS), and Altomare Obstructive Score (AOS). Repeated measures ANOVA was performed to assess the impact of surgical technique (Lap-LRR [Laparoscopic Resection Rectopexy], NOSE-RRR [Natural Orifice Specimen Extraction –Robotic Resection Rectopexy], and NOSE-LRR[Natural Orifice Specimen Extraction – Laparoscopic Resection Rectopexy]) on functional outcomes. Overall, improvement over time was observed in all groups, with the general pattern of recovery largely consistent across techniques.

Inspection of mean scores revealed observed trends. Lap-LRR patients achieved the lowest mean incontinence scores at six months, whereas NOSE-RRR patients reached the lowest ODS and constipation scores. NOSE-LRR patients showed less pronounced improvements in both continence and ODS. These trends represent non-significant differences, and the limited sample size may reduce the power to detect subtle subgroup effects. Although trends between Lap-LRR, NOSE-LRR and NOSE-RRR were observed, these differences did not reach statistical significance and should therefore be interpreted as exploratory and hypothesis-generating rather than definitive.

Overall, obstructive defecation, constipation, and fecal incontinence improved, supporting the efficacy of these interventions. Patients reported high satisfaction with the surgical outcomes. These findings align with previous studies demonstrating that rectal resection and rectopexy significantly improve constipation and obstructive defecation [17–22].

Improvement in fecal incontinence, as assessed by WIS, was more variable. Although a positive trend was observed, statistical significance was not reached. Given a significant main effect of gender, WIS was analyzed separately for male and female patients, but subgroup analyses also failed to reach significance. High variance in WIS scores and the relatively short follow-up may have contributed to the lack of statistical significance. Additionally, sphincter function may improve more gradually than captured within six months. Differences in male and female response to surgery may also influence statistical power.

Other surgical approaches, such as two-stage procedures (e.g., Laparoscopic Ventral Mesh Rectopexy [LMVR, ] combined with sigmoid resection), have shown significant improvements in incontinence over 3- and 6-month follow-up periods in our registry data, while short-term improvement at one month was non-significant [23]. However, overall functional outcomes remain mixed. Stéphane Benoist et al. found no difference between resection rectopexy, suture rectopexy, and mesh rectopexy regarding incontinence, with all three methods achieving over 75% improvement. Addition of sigmoid colon resection to laparoscopic rectopexy yielded the best results for constipation [24].

Combining single-stage LMVR with rectal wall plication (RP, Rectal Plication) improves bowel function and quality of life compared with LMVR alone [25]. Singh et al. reported in a 7-year follow-up that 69% of patients experienced improved bowel symptoms, 12% worsened, and 15% developed new pelvic pain, while 47% reported improvement [14]. M Barra et al. described a 10.5% recurrence rate at 10 years, with worse outcomes in those experiencing recurrence [15]. Jin Hidaka et al. demonstrated that after six years, constipation and incontinence outcomes favored LVMR compared with laparoscopic posterior sutured rectopexy (LPSR), with significantly lower recurrence (8.8% vs. 23%) [16]. Comparisons with vaginal rectocele repair (VRR) showed improvement in ODS scores for both LVMR and VRR, with no clear superiority [26].

In our registry, a two-stage approach combining LMVR with sigmoid resection via robot-assisted recto-sacropexy with mesh placement yielded significant improvements in constipation and ODS at six months. The rationale was that LMVR alone corrected pelvic floor depression but did not relieve obstruction from an elongated sigmoid; thus, sigmoid resection was added. Staging was also intended to reduce the risk of mesh infection [23]. Solari et al. identified dolichosigma as a predictor of persistent constipation if untreated [1] and Wang et al. confirmed the safety and efficacy of robotic and laparoscopic ventral rectopexy with colectomy for selected ODS patients [18].

Regarding ODS symptoms assessed by WCS and AOS, significant improvements were observed, with the largest changes within the first month. These improvements remained stable or slightly increased over six months. The greatest benefits were seen in patients with the highest preoperative scores, suggesting earlier surgical intervention may be advantageous. These results align with prior systematic reviews. Samaranayake et al. reported substantial reductions in fecal incontinence and constipation after colorectal resection, with weighted mean decreases of 45% and 24%, respectively, and a low recurrence rate of 3.4% [27]. Similar findings were reported by Xynos et al., Lindsey et al., and Kalev et al. [28–30]. Comparable benefits have been documented for combined LMVR and sigmoid resection, although operative times and hospital stays were longer [23].

This study also highlights differences in postoperative complications. Lap-LRR was associated with a higher incidence of Clavien–Dindo grade IIIb complications and reoperations compared with both NOSE approaches. No significant differences were observed for wound healing disorders, anastomotic leakage, or grade IVa events. A trend toward higher grade IIIa complications was noted in NOSE-LRR, though not statistically significant. These findings suggest NOSE resections may reduce severe complications and reintervention rates, supporting their safety and feasibility.

Other studies provide additional context. Kim et al. found no improvement in quality of life or functional outcomes after laparoscopic rectal resection, underscoring that not all patients benefit equally [31]. Prior surgeries may influence outcomes, as reported by Firat et al., with negative impacts of benign hysterectomy on ODS, incontinence, and constipation [32]. In our study, prior operations were recorded but not stratified by type, location, or timing, which could modify functional outcomes.

Further evidence for resection rectopexy comes from Durbeck et al., who reported significant reductions in constipation with modest improvements in continence using the KESS score [33]. Gallo et al., in a 9-year follow-up after laparoscopic Frykman–Goldberg procedures, observed sustained improvements in incontinence, constipation, and ODS [17]. Wang et al. and Kalev et al. reported similar improvements using WCS and WIS [18, 30]. Gallot et al. described improvement in incontinence after posterior Orr–Loygue rectopexy with sigmoid resection, but 38% developed new-onset constipation [34]. Rudroff et al. recently reaffirmed that combining resection rectopexy with sacrocolpopexy improves ODS, constipation, and incontinence [22].

### Limitations

This study has several limitations. First, follow-up was limited to six months, precluding conclusions on long-term outcomes or recurrence. Second, the sample size (*n* = 85) is moderate, and subgroup analyses (by surgical technique or gender) may be underpowered. Third, prior operations were not stratified by type, anatomical location, or timing, potentially influencing outcomes. Fourth, standardized quality-of-life questionnaires beyond WCS, WIS, and AOS were not applied, limiting assessment of broader patient-reported outcomes.

## Conclusion

Patients with ODS benefit significantly from rectal resection and rectopexy, with marked improvement in chronic constipation. Outcomes for fecal incontinence remain more variable. Follow-up was limited to six months; long-term functional outcomes and recurrence rates remain to be established. Further research with extended follow-up and refined assessment tools is needed to better understand factors influencing postoperative recovery. The impact of prior operations, stratified by type, location, and timing, should be further investigated to optimize patient selection and surgical outcomes. Continued research is essential to refine surgical techniques and improve functional recovery for all patients.

## Supplementary Information

Supplementary Material 1.

Supplementary Material 2.

## Data Availability

The datasets generated and analyzed during the current study are available from the corresponding author upon reasonable request.

## Abbreviations

ODS: Obstructive Defecation Syndrome
WCS: Wexner Constipation Score
WIS: Wexner Incontinence Score
AOS: Altomare Obstructive Score
NOSE: Natural Orifice Specimen Extraction
LRR: Laparoscopic Resection Rectopexy
LRR–NOSE: Laparoscopic Resection Rectopexy with NOSE
RRR–NOSE: Robotic Resection Rectopexy with NOSE
VRR: vaginal rectocele repair
LPSR: laparoscopic posterior sutured rectopexy
LMVR: Laparoscopic Ventral Mesh Rectopexy
RP: Rectal Plication
