# Supplementary material for: Minimally invasive resection rectopexy as a treatment method for obstructive defecation (ODS): functional outcome in ODS; constipation and fecal incontinence
Source: BMC Surg. 2026 Mar 5;26:330. doi: 10.1186/s12893-026-03560-5 (PMC13182115; doi:10.1186/s12893-026-03560-5)
Supplement: Supplementary file 1 — Supplementary Material 1. [file 12893_2026_3560_MOESM1_ESM.pdf]

Survey form for ODS patients

**Patient name**		**Date of birth**	
**Date of surgery**		**Age (years)**	
**Gender**	☐ Female ☐ Male	**ASA (I–VI)**	
**Weight (kg)**		**Height (cm)**	
**BMI (kg/m²)**		**Number of previous operations**	
**Diabetes mellitus**	☐ No ☐ Type 1 ☐ Type 2	**Arterial hypertension**	☐ yes ☐ no
**Constipation**	☐ yes ☐ no	**Fecal incontinence (degree)**	☐ I° ☐ II° ☐ III°
**Rectal prolapse (grade I–IV)**		**Rectocele**	☐ Yes ☐ No
**Intussusception (grade I–IV)**			
**Operating time (min)**		**Conversion**	☐ Yes ☐ No
**Time to flatus (h)**		**Time to defecation (h)**	
**Wound healing disorder**	☐ yes ☐ no	**Pain (NRS 0–10)**	
**Anastomotic leakage**	☐ yes ☐ no	**Reoperation**	☐ yes ☐ no
**Clavien-Dindo (grade I–V)**		**Which complication**	
**Length of hospital stay (days)**		**Satisfaction**	☐ yes ☐ no

Scores from the original questionnaires Wexner Constipation Score **(WCS),** Wexner Incontinence Score **(WIS)** and Altomare Obstructive Defecation Syndrome **(AOS)** Score;

**Score**	**preOP**	**1M**	**3M**	**6M**	**GM**	**12M**
**Constipation score**						
**ODS score**						
**Incontinence score**						

Pre-op = baseline assessment (within 4 weeks prior to surgery).
