# Supplementary material for: Minimally invasive resection rectopexy as a treatment method for obstructive defecation (ODS): functional outcome in ODS; constipation and fecal incontinence
Source: BMC Surg. 2026 Mar 5;26:330. doi: 10.1186/s12893-026-03560-5 (PMC13182115; doi:10.1186/s12893-026-03560-5)
Supplement: Supplementary file 2 — Supplementary Material 2. [file 12893_2026_3560_MOESM2_ESM.docx]

| **Wexner constipation score** | |  | **ODS score according to Altomare** | |  |  |
| --- | --- | --- | --- | --- | --- | --- |
| **Frequency of bowel movements** | **Score** |  | **Time: minutes spent on the toilet per attempt** | **Score** |  |  |
| 1-2 times every 1-2 days | 0 |  | Up to 5 minutes | 0 |  |  |
| 2 times per week | 1 |  | 6-10 | 1 |  |  |
| Once a week | 2 |  | 11-20 | 2 |  |  |
| Less than once a week | 3 |  | 21-30 | 3 |  |  |
| Less than once a month | 4 |  | More than 30 | 4 |  |  |
| **Difficulty: Painful emptying** | **Score** |  | **Attempts to empty bowels per day?** | **Score** |  |  |
| Never | 0 |  | 1 | 0 |  |  |
| Rarely | 1 |  | 2 | 1 |  |  |
| Sometimes | 2 |  | 3 – 4 | 2 |  |  |
| Often | 3 |  | 5 – 6 | 3 |  |  |
| Always | 4 |  | > 6 | 4 |  |  |
| **Completeness: Feeling of incompleteness** | **Score** |  |  | **Score** |  |  |
| **Emptying** |  |  | **How often do you use your hand or finger to enable defecation?** |  |  |  |
| Never | 0 |  | Never | 0 |  |  |
| Rarely | 1 |  | Once a month to once a week | 1 |  |  |
| Sometimes | 2 |  | Once a week | 2 |  |  |
| Often | 3 |  | 2–3 times per week | 3 |  |  |
| Always | 4 |  | Always | 4 |  |  |
| **Failure: Unsuccessful attempts to empty the bladder** | **Score** |  | **Do you take laxatives?** | **Score** |  |  |
| **/24 hours** |  |  | Never | 0 |  |  |
| Never | 0 |  | Once a month to once a week | 1 |  |  |
| 1-3 | 1 |  | Once a week | 2 |  |  |
| 3-6 | 2 |  | 2–3 times per week | 3 |  |  |
| 6-9 | 3 |  | Always | 4 |  |  |
| More than 9 | 4 |  | **Do you use enemas? (Klysma)** | **Score** |  |  |
| **Pain: Abdomen** | **Score** |  | Never | 0 |  |  |
| Never | 0 |  | 1x per month to 1x per week | 1 |  |  |
| Rarely | 1 |  | Once a week | 2 |  |  |
| Sometimes | 2 |  | 2–3 times a week | 3 |  |  |
| Often | 3 |  | Always | 4 |  |  |
| Always | 4 |  | **Is emptying incomplete?** | **Score** |  |  |
| **Time: minutes spent on the toilet per attempt** | **Score** |  | Never | 0 |  |  |
| Less than 5 | 0 |  | 1x per month to 1x per week | 1 |  |  |
| 5-10 | 1 |  | Once a week | 2 |  |  |
| 10-20 | 2 |  | 2–3 times per week | 3 |  |  |
| 20 | 3 |  | Always | 4 |  |  |
| More than 30 | 4 |  | **Do you have to strain hard when having a bowel movement?** | **Score** |  |  |
| **Medical history: Duration of constipation (years)** | **Score** |  | Never | 0 |  |  |
| 0 | 0 |  | Once a month to once a week | 1 |  |  |
| 1-5 | 1 |  | Once a week | 2 |  |  |
| 5-10 | 2 |  | 2–3 times per week | 3 |  |  |
| 10 | 3 |  | Always | 4 |  |  |
| More than 20 | 4 |  | **How is the stool?** | **Score** |  |  |
| **Support: Type of support** | **Score** |  | Soft | 0 |  |  |
| No support | 0 |  | hard | 1 |  |  |
| Stimulant laxatives | 1 |  | Hard and little | 2 |  |  |
| Digital support/occasional enema | 2 |  | Like stone | 3 |  |  |
| Digital support/enema always | 3 |  |  |  |  |  |
|  |  |  |  |  |  |  |
| **Points (total) ………………/31** | |  | **Points (total) ………………/31** | |  |  |

| **Wexner incontinence score** |  |
| --- | --- |
| How often do you lose control of your bowel movements? | **Score** |
| Never | 0 |
| Less than once a month | 1 |
| more than once a month | 2 |
| More than once a week | 3 |
| mostly daily | 4 |
| How often do you have uncontrolled loose stools? | **Score** |
| Never | 0 |
| Less than once a month | 1 |
| More than once a month | 2 |
| More than once a week | 3 |
| mostly daily | 4 |
| How often do you involuntarily pass wind? | **Score** |
| Never | 0 |
| Less than once a month | 1 |
| More than once a month | 2 |
| More than once a week | 3 |
| mostly daily | **4** |
| How often do you wear a pad? | **Score** |
| Never | 0 |
| Less than once a month | 1 |
| More than once a month | 2 |
| More than once a week | 3 |
| mostly daily | 4 |
| How often do you have to go to the doctor because of stool problems? | **Score** |
| change your regular lifestyle habits? |  |
| Never | 0 |
| Less than once a month | 1 |
| More than once a month | 2 |
| More than once a week | 3 |
| mostly daily | 4 |
|  |  |
| **Points (total) ………………/20** |  |
|  |  |
|  |  |
|  |  |
|  |  |
|  |  |
|  |  |
|  |  |
|  |  |
|  |  |
|  |  |
|  |  |
|  |  |
|  |  |
|  |  |
|  |  |
|  |  |
|  |  |
